# Remote Positioning of Spherical Alginate Ferrogels in a Fluid Flow by a Magnetic Field: Experimental and Computer Simulation

**DOI:** 10.3390/gels9090711

**Published:** 2023-09-01

**Authors:** Felix Blyakhman, Alexander Safronov, Ilya Starodumov, Darya Kuznetsova, Galina Kurlyandskaya

**Affiliations:** 1 Department of Biomedical Physics and Engineering, Ural State Medical University, Ekaterinburg 620028, Russia; ilya.starodumov@urfu.ru (I.S.); ash--2000@mail.ru (D.K.); 2 Institute of Natural Sciences and Mathematics, Ural Federal University, Ekaterinburg 620002, Russia; alexander.safronov@urfu.ru (A.S.); galinakurlyandskaya@urfu.ru (G.K.); 3 Institute of Electrophysics UB RAS, Ekaterinburg 620016, Russia

**Keywords:** biopolymer, alginate gel, magnetic particles, spherical ferrogel, delivery system, magnetic field, fluid flow, biomedical applications

## Abstract

This work belongs to the development of mechanical force-responsive drug delivery systems based on remote stimulation by an external magnetic field at the first stage, assisting the positioning of a ferrogel-based targeted delivery platform in a fluid flow. Magnetically active biopolymer beads were considered a prototype implant for the needs of replacement therapy and regenerative medicine. Spherical calcium alginate ferrogels (FGs)~2.4 mm in diameter, filled with a 12.6% weight fraction of magnetite particles of 200–300 nm in diameter, were synthesized. A detailed characterization of the physicochemical and magnetic properties of FGs was carried out, as were direct measurements of the field dependence of the attractive force for FG-beads. The hydrodynamic effects of the positioning of FG-beads in a fluid flow by a magnetic field were studied experimentally in a model vessel with a fluid stream. Experimental results were compared with the results of mathematical and computer modeling, showing reasonable agreement. The contributions of the hydrodynamic and magnetic forces acting on the FG-bead in a fluid flow were discussed. Obtained forces for a single ferrogel implant were as high as 0 to 10^−4^ N for the external field range of 0 to 35 kA/m, perfectly in the range of mechanical force stimuli in biological systems.

## 1. Introduction

The creation of smart drug delivery systems with a high level of drug release control at the desired position, at a planned time point, and with the ability to respond either to physiological or external stimuli is in the focus of modern biomedicine [1,2,3]. Among the wide variety of targeted delivery systems, the mechanical force-responsive systems are of great interest [4]. Such systems provide for the release of therapeutic substrates through controlled mechanical stimuli such as compression, tension, or shear. In particular, the hydrogels are mainly suitable materials for drug release by compression [5].

Hydrogels synthesized by gelation of biopolymers such as polysaccharides like alginate or structural proteins like actin are introduced for biomedical applications due to their high biocompatibility [6,7,8]. In particular, alginic acid hydrogels are widely used for needs in cellular technologies, tissue engineering, drug delivery, etc. [9,10,11,12,13,14,15].

The addition of magnetic particles in the course of the alginate hydrogel synthesis forms stable composites—alginate ferrogels (FGs) [16,17,18]. In general, FGs are magnetically active soft materials, and, in the presence of an external magnetic field, they demonstrate a magneto-deformation effect and/or mechanical motion along magnetic field lines [19,20,21]. Both of these features expand the scope of FGs use in biomedicine [22,23,24]. In particular, alginate FGs are considered magnetically controlled platforms for tissue engineering and targeted delivery of therapeutic substances, including implants for replacement therapy [25,26,27]. In addition, ferrogel beads filled with superparamagnetic nanoparticles such as polystyrene composite spheres [28,29] will not interact and aggregate in a zero external field but obtain a reasonably high magnetic moment under the application of the field. Both features can be used in magnetic separation and magnetic biosensing [30,31].

The present study belongs to the direction of the development of mechanical force-responsive drug delivery systems based on remote stimulation by an external magnetic field at the first stage, assisting the ferrogel bead delivery, and at the second stage, acting as a magnetic trap. Spherical calcium alginate ferrogels (FGs)~2.4 mm in diameter, filled with a 12.6% weight fraction of magnetite particles of 200–300 nm in diameter, were considered a prototype of a targeted delivery implant for the needs of replacement therapy and regenerative medicine in hollow organs. This concentration was selected taking into account existing experience with the magnetic and microwave properties of filled composites [32]. The most general rule is that for 5 wt.% of magnetic filler, one can consider the system as a system of non-interacting MNPs. 12 wt.% present is still close to the above-mentioned limit, but the bead with 12.6 wt.% iron oxide concentration provides the higher magnetic moment for each bead and therefore the higher force calculated for one bead.

First, alginate gel was chosen due to its excellent biocompatibility and wide application in cellular technologies, as noted above. In addition, the synthesis of spherical calcium alginate gels is well described in the literature and is quite easy to perform [18]. Biocompatible magnetite particles of iron oxide are also well studied and widely introduced as a filler for FGs. In particular, in this work, the same particles were used as in our recent study on the interaction of ferrogels with a magnetic field [33].

In Ref. [33], a methodological approach to directly determining the attractive force of FGs on a magnet was reported for the first time. Furthermore, a mathematical model to calculate this force based on the magnetic properties of FG and magnets was also proposed. In that investigation [33], cylindrical composite FGs containing polyacrylamide, polysaccharides, and magnetite microparticles were studied.

In this work, we applied a similar methodology for spherical FGs to realize the universality of the proposed approach, on the one hand. On the other hand, this approach was applied to study the mechanical behavior of an FG-bead in the stream of a fluid flow in the presence of a magnetic field. For this purpose, the original experimental setup was designed, and a mathematical model was also proposed. Thus, the present work is the first attempt to quantitatively describe the balance of hydrodynamic and magnetic forces for the remote positioning of a spherical delivery system in a fluid flow.

Here, we present the results of detailed characterization of the physicochemical and magnetic properties of FGs as well as direct measurements of the field dependence of the attractive force for FG beads. With the use of an experimental model of a vessel with a fluid, the hydrodynamic effects of the positioning of FG-beads in a fluid flow by a magnetic field are considered. The comparison analysis of experimental results with the results of mathematical and computer modeling is present as well. The contributions of the hydrodynamic and magnetic forces acting on the FG-bead in a fluid flow are the focus of discussion.

## 2. Results and Discussion

**Figure 1:**
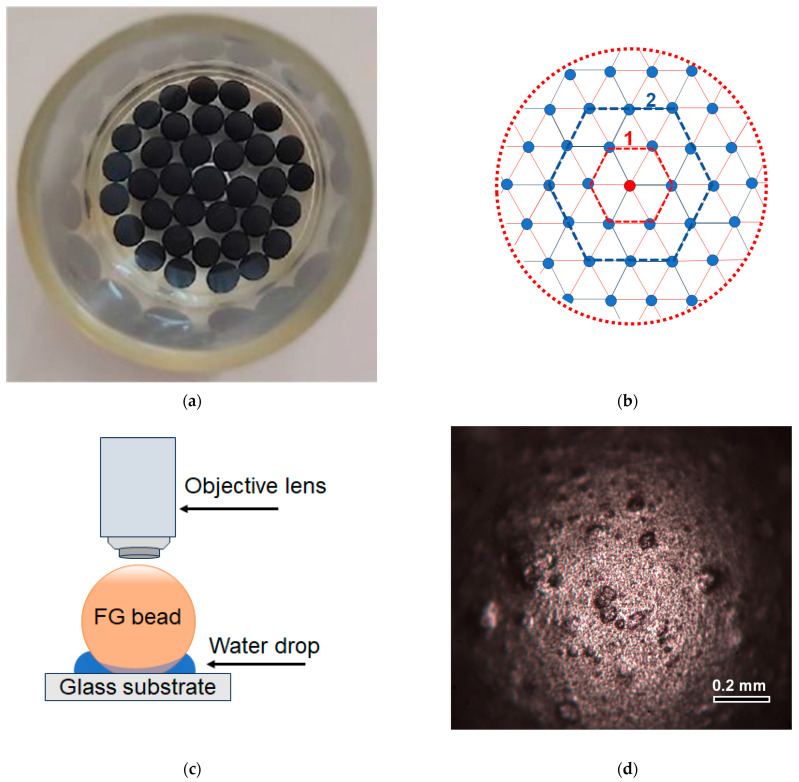
Top view of the ferrogel beads under the study (**a**). General arrangement of identical superparamagnetic spheres self-organized as one-layer bead arrays placed onto flat surfaces: 1—line for indication of nearest neighbors corresponding to central red bead; 2—the second level of the neighbors corresponding to central red bead. Red dashed line indicates the bead cluster corresponding to experimental one (**b**). Description of the positioning of ferrogel spherical sample for observation of the surface properties by optical microscopy (**c**). Optical microscopy image of the surface of FG bead (**d**).

The study was performed on spherical beads of composite ferrogels based on a physical network of calcium alginate (CaAlg) filled with magnetite particles. For the force measurements, beads were arranged as one-layer bead arrays placed onto a flat surface of an appropriate Petri dish (Figure 1a). The average diameter of ferrogel beads was 2.37 mm, with a standard error of 0.12 mm (*n* = 100). Figure 1b shows the ideal re-arrangement of identical superparamagnetic spheres self-organized as one-layer beads arrayed onto a flat surface. Previously, the behavior of similar arrays perpendicular or parallel to the substrate’s external magnetic field was studied for commercial spheres, Dynabeads^®^ M-480, for their different concentrations (Dynal Biotech ASA, Oslo, Norway) [34]. To some extent, their behavior is similar to the observed behavior of the ferrogel beads under consideration. However, the Dynabeads^®^ M-480 can be viewed as an infinite structure due to its small size (about 5 μm in diameter) and the large number of beads forming the array.

Under the application of the external magnetic field perpendicular to the substrate, Dynabeads^®^ M-480 were forming a hexagonal, densely packed structure. Below, we will use some parameters of the geometrical bead arrangements in order to estimate the degree of their interaction under the application of the external magnetic field.

### 2.1. Characterization of Magnetic Particles

**Figure 2:**
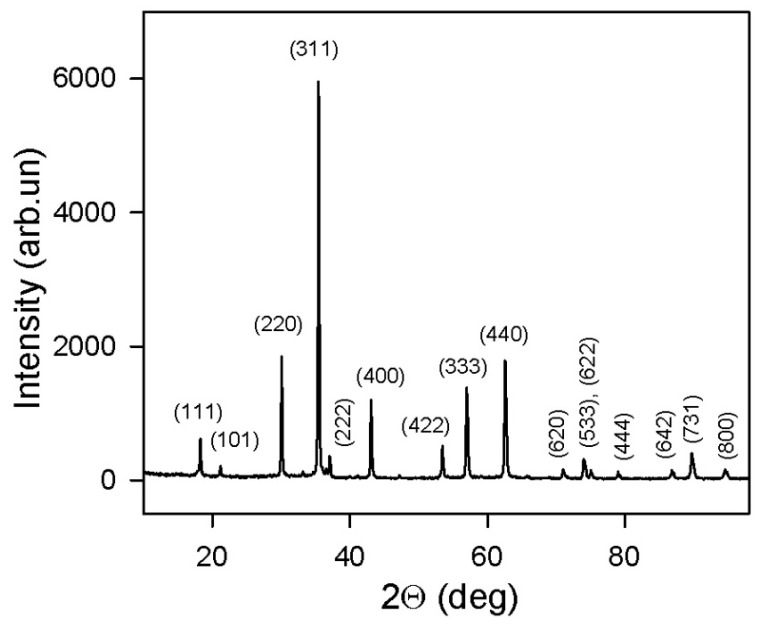
XRD pattern for magnetite particles used in the preparation of ferrogel beads. Numbers correspond to Miller indexes.

**Figure 3:**
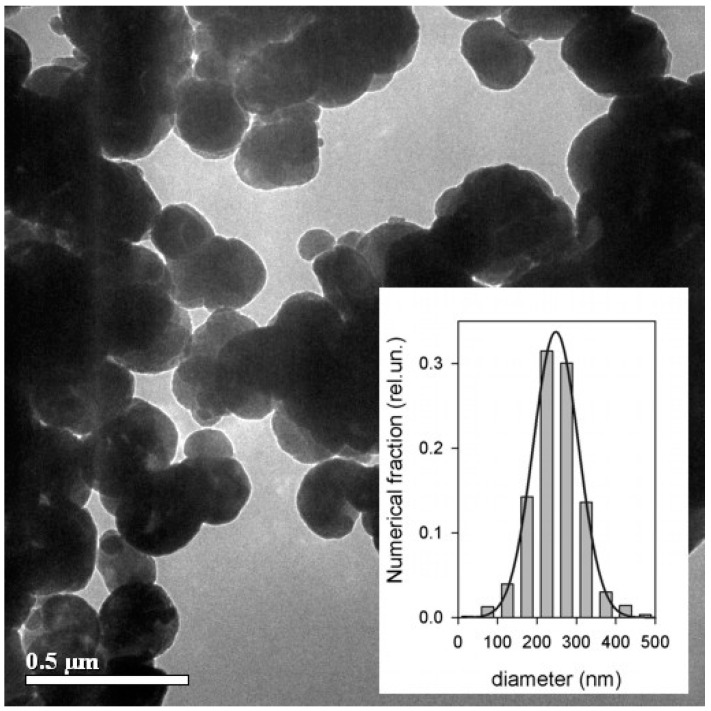
TEM images of magnetite particles. The inset gives numerical particle size distribution (PSD) calculated by the analysis of 632 images.

Magnetite (Fe_3_O_4_) was a commercial product purchased from Alfa Aesar (Alfa Aesar, Ward Hill, MA, USA). The X-ray diffraction (XRD) pattern given in Figure 2 corresponded to Fe_3_O_4_ phase—94%, Fe_2_O_3_ phase—1%, and FeO(OH) phase—5% (wt). Transmission electron microscopy (TEM) microphotographs of magnetite particles are given in Figure 3.

The particles were quasi-spherical, with caliper sizes in the range of 50–500 nm. The numerical particle size distribution (PSD) of magnetite (Figure 3, inset; image analysis of 632 particles) was well fitted by a Gaussian distribution function with a median of 248 nm and a dispersion of 57 nm. The weight average caliper diameter (D_w_) determined via PSD was found to be 305 nm. Typically, the PSD of powdered solids is rather lognormal, but for that specific batch of commercial magnetite, a Gaussian distribution gave a much better fit in the 0–500 nm range of particle caliper size.

Noteworthy, the XRD diffractogram can give information on the average size of coherent domains in the crystalline structure of particles using the Sherer equation. It gave 90 nm for magnetite MPs. This value is lower than the average caliper diameter of particles calculated from TEM images. It means that some of the magnetite MPs were polycrystalline.

**Figure 4:**
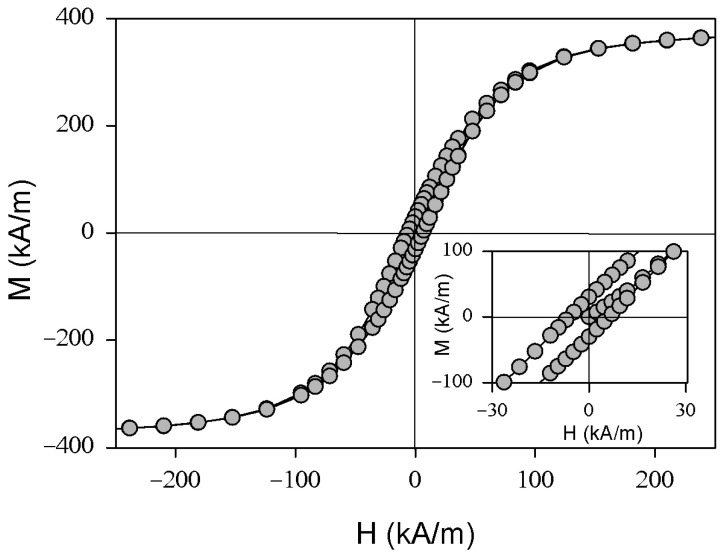
Magnetic hysteresis loops for magnetite particles. M—magnetization; H—magnetic field strength. Insert reflects part of the hysteresis loops close to zero fields, representing the low external magnetic field range at which the primary magnetization curve becomes better visible.

Saturation magnetization of magnetite particles (Figure 4) was 388 kA/m, remnant magnetization was 30 kA/m, and coercivity was 6.2 kA/m. The average size of the particles estimated using XRD and TEM data indicates that the magnetic particles under consideration are not in a superparamagnetic state, i.e., they have remnant magnetization and non-zero coercivity at room temperature [35,36,37]. From the point of view of biomedical applications, the most desired combination of magnetic properties is a zero magnetic moment in zero externally applied field and a rather high magnetic moment in fields below 2 T, as approved by biomedical regulations [38]. In the present study, we use commercial magnetite, for which the analysis of the shape of magnetic hysteresis loops and comparison with the data reported in the literature confirm that we are dealing with magnetite having the properties of bulk material [29,30,31] with high saturation magnetization and coercivity. In the nanoscale, the saturation magnetization of the magnetite depends on the average size and the oxidation degree of the iron oxide [37,39]. However, for the small particles, the rule that the larger the particle, the higher the saturation magnetization, is well established. In our particular case, it is possible to obtain the value of the magnetic moment of the FG bead (as the mass m is known) in each applied magnetic field. Later, it will be seen that the obtained FG beads have a high enough total magnetic moment to create an attraction force sufficient for control of the bead trajectory.

### 2.2. Characterization of Ferrogels

Figure 1c,d show the optical microscopy image of the surface of a single FG bead placed symmetrically with respect to the objective lens. Due to the rather limited focal range of the optical microscope, one can see with high resolution only the top area. However, it clearly confirms the degree of sphericity of the bead and indicates the approximate size of the surface defects (below 0.1 mm in diameter).

Equilibrium swelling ratios of CaAlg ferrogel (FG) and blank CaAlg hydrogel (HG) were calculated according to the following equation:

(1) $$\alpha =\frac{m-m_{0}}{m_{0}},$$

here *α* is the equilibrium swelling ratio, *m* is the mass of the swollen gel, *m*_0_ is the mass of the dried gel.

The integral swelling ratios of HG and FG are given in Table 1, and their values differ substantially—approximately three-fold. This is because the dry residue of FG contained solid magnetite particles, which also count in the calculation of the swelling ratio, although the water uptake is provided exclusively by the polymeric network of CaAlg. To determine the swelling ratio of the polymeric matrix, magnetite particles are to be excluded from consideration. The first step was to determine their content in dry FG by thermal analysis.

**Figure 5:**
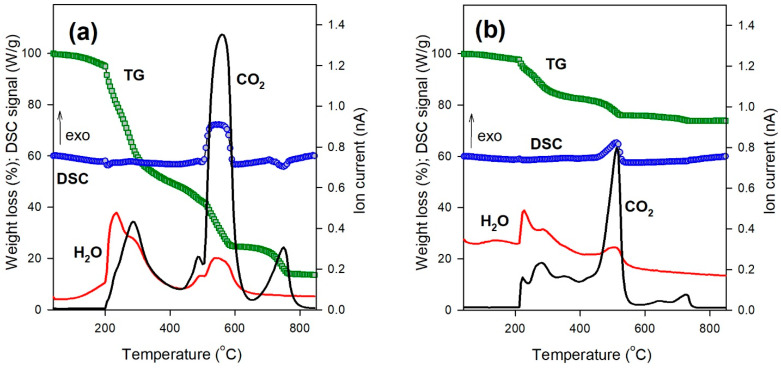
Thermal analysis plots for CaAlg dried hydrogel (**a**) and ferrogel (**b**). The green line (TG) shows the mass loss of the sample. The blue line (DSC) corresponds to the thermal effect of decomposition. The red (H_2_O) and black (CO_2_) lines correspond to the mass spectral signal proportional to the water and carbon dioxide content released in decomposition.

Figure 5 presents the characteristic curves of thermogravimetry (TG), differential scanning calorimetry (DSC), and quadrupole mass spectrometry (QMS) recorded upon heating dry HG (Figure 5a) and FG (Figure 5b) samples from 50 to 850 °C.

The thermal decomposition of HG and FG included three distinct steps in the TG thermogram. The first one is in the temperature range 200–300 °C, the second is at 500–600 °C, and the third is at 700–800 °C. The first and second steps were accompanied by the evolution of both water (H_2_O) and carbon dioxide (CO_2_). In the third step, only the release of CO_2_ was observed. The major exothermic heat effect was observed at the second step of CaAlg decomposition. The heat effect at the first step was very small. In the third step, the endothermal effect of heat absorption was observed for HG decomposition.

The mechanism of thermal decomposition of polymers is, in general, very complicated, and its detailed analysis is beyond the scope of the present study. Nevertheless, it seems reasonable that the first step of CaAlg decomposition at 200–300 °C might be associated with the formation of volatile organic products of thermolysis. These compounds burned down to H_2_O and CO_2_ not in the crucible at the DSC holder but in the gas phase. Therefore, the weight loss step at the TG thermogram was the largest, but the DSC signal from this process was very weak. The peaks of H_2_O and CO_2_ evolution at QMS thermograms were comparable at this stage. In the second step, at 500–600 °C, thermolysis occurred on non-volatile carbonized polymeric residues in the DSC crucible. Therefore, the recorded exothermal heat effect was large. The peak of CO_2_ evolution was substantially larger than that of H_2_O. The third step of CaAlg destruction is related to the decomposition of Ca carbonate into Ca oxide. The process was endothermal, and the only volatile product was CO_2_.

The appearance of the thermograms for CaAlg HG and FG was very much the same. The difference was only in the absolute values of the effects. This difference was used for the evaluation of magnetite content in dried FG beads. In the case of HG, the residue after decomposition contained only Ca oxide; in the case of FG, it was a mixture of Ca oxide and magnetite particles, which remained intact during heating at these conditions. The maximal weight losses for HG and FG dried gels are given in Table 1. Based on their values, the content of magnetite in dried FG was calculated using the following equation:

(2) $$\gamma =1-\frac{\Delta_{FG}}{\Delta_{HG}}$$

Here, *γ* is the weight fraction of magnetite in dried FG, and ∆*_HG_* and ∆*_FG_* are integral weight losses at the thermal decomposition of HG and FG, respectively. The value of *γ* is given in Table 1. Using this value, the actual swelling ratio of CaAlg polymeric matrix (*a*’) and the weight fraction of magnetite in swollen FG (*w*) were calculated according to the equations [39]:

(3) $$\alpha ’=\frac{\alpha }{1-\gamma }$$

and

(4) $$\omega =\frac{\gamma }{1+\alpha }$$

where *α* is the equilibrium swelling ratio.

It can be seen from Table 1 that the actual swelling ratio of the CaAlg network in FG was almost the same as for CaAlg HG. The weight content of magnetite in FG (12.6%) was a little higher than that in the suspension of magnetite (10%) in NaAlg solution for the preparation of FG beads. Likely, it is the consequence of the slight contraction of droplets during their physical gelation by Na^+^/Ca^2+^ exchange in the synthesis.

Based on the value of magnetite content in FG beads, their magnetic susceptibility was calculated using the calibration presented in our recent paper [33].

(5) $$\chi_{FG}=\chi_{EC}\frac{\varphi_{FG}}{\varphi_{EC}}$$

Here, *χ_FG_* is the susceptibility of FG, *φ_FG_* is the volume fraction of magnetite in FG. *χ_EC_* and *φ_EC_* are the susceptibility and volume fraction of magnetite of the reference spherical bead of epoxy composite with 30% (wt.) magnetite content. In the present study, we have used the same batch of magnetite as in Ref [33], and we could use the calibration values *χ_EC_* = 0.408 and *φ_EC_* = 9.57% as well. Calculation according to Equation (5) gave *χ_FG_* = 0.13. This value is given in Table 1.

### 2.3. Effect of Magnetic Field Strength on the Attractive Force of Ferrogel Beads to a Magnet

To determine the attractive force of the ferrogel beads on the core of the electromagnet (EM), an experimental setup was used (see [33] and the methodological section below). The common attractive force of 36 beads located in one layer at the bottom of the cuvette filled with water (see Figure 1) was evaluated. The cuvette diameter was commensurate with the core of EM. Various strengths of a constant magnetic field were discretely set by the application of a constant electric voltage to the coil of EM. The measurements were carried out on three different sets of beads. For each set, three repetitions were performed.

**Figure 6:**
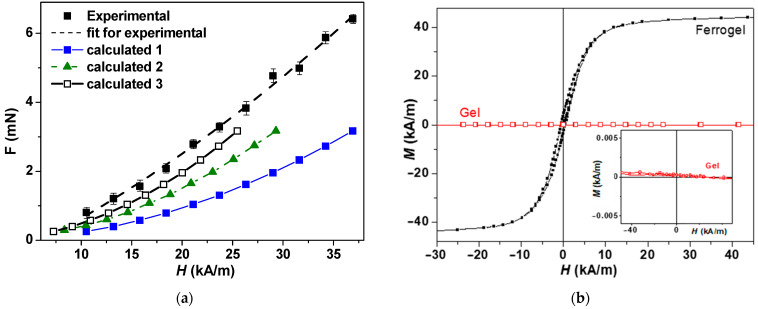
(**a**) Dependence of the attractive force (*F*) in ferrogels on the magnetic field strength (*H*) for 36 magnetic beads. The confidence interval for *F* is given at *p* = 0.05 (n = 9): y = 0.00002x^2^ + 0.0044x for the fit of the experimental curve. 1—calculations in accordance with Equation (6) (case of non-interacting beads); 2—calculations in accordance with Equation (6) but corrected for dipole-dipole interactions taking into account the first group of nearest neighbors; 3—correction of case 2 for possible change of primary magnetization curve taking into account interactions with the first group of nearest neighbors; 4—calculations in accordance with Equation (6) but corrected for dipole-dipole interactions taking into account the second group of nearest neighbors (see also Figure 1b). (**b**) Magnetic hysteresis loop of gel and ferrogel beads; inset shows the hysteresis loop of the blank gel bead in the scale, making the slope of the magnetization curve visible.

Figure 6a shows the dependence of the attractive force in ferrogel on the magnetic field strength obtained for 36 samples. One can see that the gradual increase in the magnetic field strength is accompanied by an increase in the attractive force of the ferrogel beads. The dependence is well approximated by a quadratic function of the second order. The value of the attractive force per sample was obtained as a quotient of the registered force divided by the number of samples (36 spheres). At field values of 21.5, 29.4, and 37.4 kA/m, the force was equal to 0.08 ± 0.003, 0.132 ± 0.007, and 0.178 ± 0.006 mN, respectively.

Previously, we described the model for the calculation of the attractive force for one gel sample (Equation (6)) [33].

(6) $$F=\mu_{0}\frac{\chi }{1+N\chi }\frac{V}{h}\frac{H_{2}^{2}-H_{1}^{2}}{2}.$$

Here $\mu_{0}$—is the magnetic permeability of vacuum, *χ* is the magnetic susceptibility, and *N* stands for the demagnetization factor. This factor was evaluated and tabulated in reference [40]. *H*_1_ and *H*_2_ stand for the field intensity at the upper and bottom faces of the ferrogel bead. Magnetic susceptibility can be estimated from the measurements of the magnetic hysteresis loop of the ferrogel bead (Figure 6b). In addition, the measurements of the blank gel showed a measurable but negligible contribution from the gel matrix in comparison with the filler.

**Figure 7:**
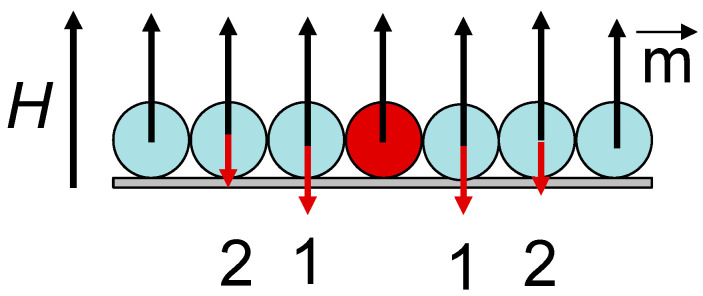
Geometry of magnetic fields for a central line of the beads in beads array (central line, Figure 1b); 1—the first type neighbors, 2—the second type neighbors. Red arrows indicate magnetic fields created by the central bead for the positions of the other beads.

According to the calculations of the attractive force using Equation (6), the force turned out to be much lower (curve 1 for Figure 6) in comparison with the experimentally obtained values. However, it is understandable because in this simple model, we did not take into account the dipole-dipole interactions between the beads that appear under the application of the external fields. Let us consider, although it is a strong suggestion that the beads are closely situated to each other, that distances are sufficient for the description of the beads interactions as the dipole-dipole type. Figure 7 describes the field H applied for the force measurement case. Ferrogel bead geometries of the magnetic fields in the system of bead arrays. External magnetic fields, under the application of an external field H, obtain equal magnetic moments m. Red arrows indicate magnetic fields created by the central bead for the positions of the other beads. This additional field H^*^ is proportional to 1/d^3^ for the neighbors of the first type and 1/8d^3^ for the neighbors of the second type. Making corrections to the effective field for the nearest neighbors of the first type, one obtains curve 2, and corrections for the contributions of the nearest neighbors of the first and second types result in the behavior described by curve 3.

Thus, the results of measurements and predictions by the model are in good agreement.

### 2.4. Effect of Magnetic Field Strength on the Positioning of Ferrogel Beads in a Fluid Flow

Experimental modeling of the mechanical behavior of a ferrogel bead in a fluid flow under the action of a magnetic field was performed using the hydrodynamic setup described in the Methods section (see below). The problem was to determine the magnitude of the magnetic field required to fix the bead on the tube wall at a given flow rate. The fluid flow rate was chosen in such a way that the ferrogel bead moved in the stream in the tube rather than rolling along its wall.

**Figure 8:**
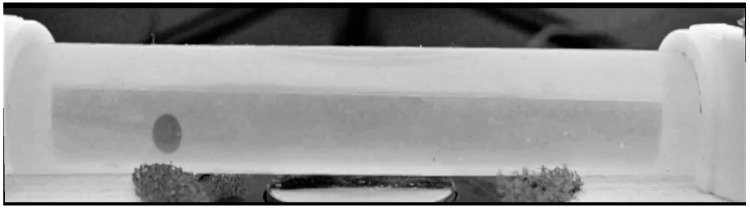
Side view of a fragment of the experimental setup for positioning the ferrogel bead in a magnetic field. The diameter of the bead is about 2.4 mm. See explanation in the text.

Figure 8 presents one frame of the video of the bead motion in the water flow. One can see a fragment of a tube with a magnetic bead, as well as the upper part of the EM right under the tube. The metal core of the EM is pressed directly against the outer wall of the tube. Fragments of foam rubber are also visible on the outer surface of the EM. This material was used as a gasket to fix the EM in the mounting hole.

Two movies reflecting the mechanical behavior of the bead at the same volume flow rate of 400 mL/s but at different values of the magnetic field strength are presented in the Supplementary Materials. In particular, Video S1 corresponds to 32 kA/m at the core of EM, and Video S2—40 kA/m. Movies reflect the bead motion with a 40 times slowdown during playback. One can see that at the lowest field strength, the velocity of bead motion at the left border of the EM core slows down, then it falls on the tube wall and rolls along it. The velocity of the bead at the right border of the EM slows down almost to zero, but the acting magnetic force is not enough to fix it on the tube wall. At the highest field strength, the deceleration of the bead at the left boundary of the EM is clearly visible, and this results in the fixation of the bead to the tube wall close to the right boundary of the EM core.

**Figure 9:**
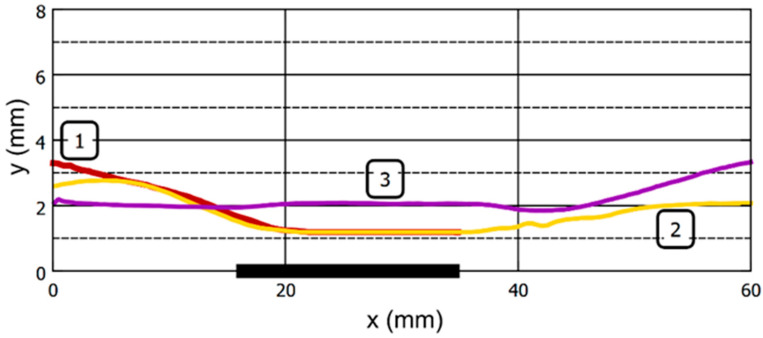
Effect of the magnetic field strength on the spatial position of the ferrogel bead in the tube at a fluid flow rate of 400 mL per sec. Tracks 1, 2, and 3 correspond to the magnetic field strengths at the surface of the EM core of 40, 32, and 24 kA/m, respectively. The origin of the coordinates corresponds to the beginning of the observation. The thick horizontal line at the X-axis represents the position of the electromagnet core. See explanation in the text.

**Figure 10:**
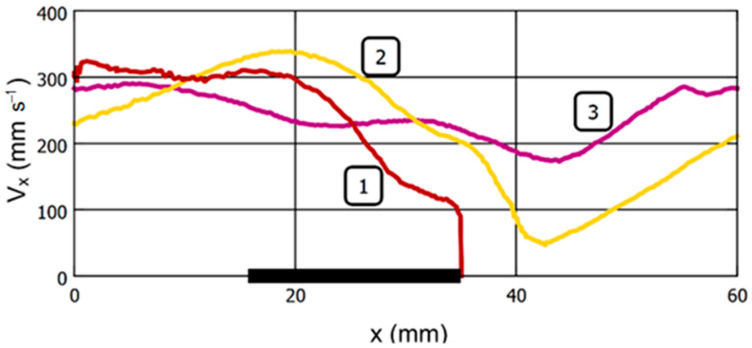
Effect of the magnetic field strength on the linear velocity of the ferrogel bead in the tube at fluid flow of 400 mL per sec. Tracks 1, 2, and 3 correspond to the magnetic field strength at the surface of the EM core of 40, 32, and 24 kA/m, respectively. The origin of the coordinates corresponds to the beginning of the observation. The thick horizontal line at the X-axis represents the position of the electromagnet core. See explanation in the text.

Figure 9 and Figure 10 illustrate the mechanical behavior of the bead for Videos S1 and S2 in graphical form. These graphs additionally provide information on the parameters of the bead motion at a magnetic field strength of 24 kA/m, at which the action of the magnetic field on the sample was not visually observed.

Figure 9 shows the spatial position of the bead detected at various points in time from the beginning of the video recording (see Videos S1 and S2). The *X*-axis reflects the position of the sample along the long axis of the tube, while the *Y*-axis presents the distance from the inner surface of the tube to the center of the bead.

Figure 10 shows the dependence of the linear velocity of a bead in a fluid flow on its position along the *X*-axis. It is noteworthy that despite the absence of visible effects of the EM on the bead at the magnetic field strength of 24 kA/m, the velocity of the sample in the area of the EM core also decreases to a certain extent.

### 2.5. Computer Simulation of Ferrogel Beads Motion in a Fluid Flow in the Presence of a Magnetic Field

**Figure 11:**
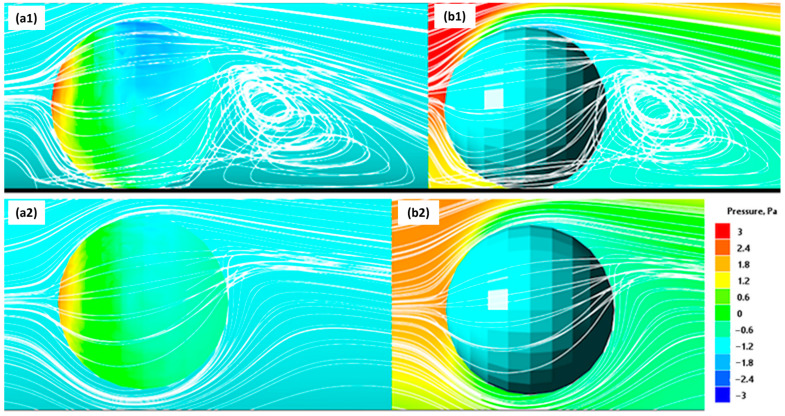
Color plot of pressure distribution at the surface and around the ferrogel sample in contact with the wall (**a1**,**b1**) and in the presence of a gap between the sample and the tube wall (**a2**,**b2**). White lines represent the streamlines of the liquid.

Figure 11 shows a longitudinal section of the tube with a spherical sample 2.38 mm in diameter and the pressure distribution around it arising in an oncoming fluid flow with a velocity of 0.35 m/s. The calculations were performed using the model described in the Methods section and reflect a hydrodynamic force acting on the sample near the wall of the tube.

Figure 11(a1,b1) show the pressure distribution at the surface of the bead (a1) and at the water surrounding the sample (b1) for the case when the bead is in contact with the tube wall (black line). One can see that the pressure patch in the front hemisphere of the bead is shifted upwards, while behind the sample, a zone of low pressure appears. Both of these factors determine the tendency for the bead to roll along the vessel wall. At the same time, the pressure in the lower part of the hemisphere significantly exceeds the pressure in the upper part of the bead (vertical pressure gradient). This causes the appearance of a lifting force that tends to move the sample along the axis of symmetry of the vessel. The streamlines also illustrate a significant vortex formation behind the ball, which is associated with a local pressure gradient that causes the flow to separate the bead from the wall.

Figure 11(a2,b2) show the results of similar hydrodynamic calculations for the case of a small gap between the bead and the vessel wall. One can see that the pressure field has qualitatively changed. The pressure spot on the front hemisphere of the sample is shifted to the center, and now the hydrodynamic force provides mainly translational motion to the bead. Also, the pressure gradient along the vertical axis is decreased significantly, which means that the lift force is reduced.

Figure 11(b2) illustrates the decrease in pressure gradients in the fluid around the bead, resulting in almost symmetrical flow around it. The streamlines also demonstrate a smooth flow around the sample without both the flow separation and the vortex formation.

The obtained results of the simulation (Figure 11) mainly reflect the effect of the vessel wall on the pressure and flow velocity around the bead. Since the size of the bead and the diameter of the vessel have commensurate values, they interact significantly with each other through the liquid. Therefore, this phenomenon must be taken into account when modeling the mechanical behavior of the ferrogel bead in the presence of a magnetic field.

**Figure 12:**
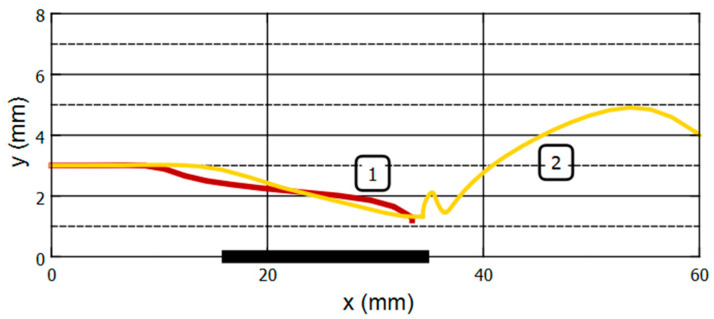
Computer simulation of the spatial position of the ferrogel bead in the tube at fluid flow using the model (the model description is presented in Section 4.5; see also Supplementary Videos S3 and S4). Tracks 1 and 2 correspond to the magnetic field strengths at the surface of the EM core of 40 and 32 kA/m, respectively. The origin of the coordinates corresponds to the beginning of the observation. The thick horizontal line at the X-axes represents the position of the electromagnet core.

**Figure 13:**
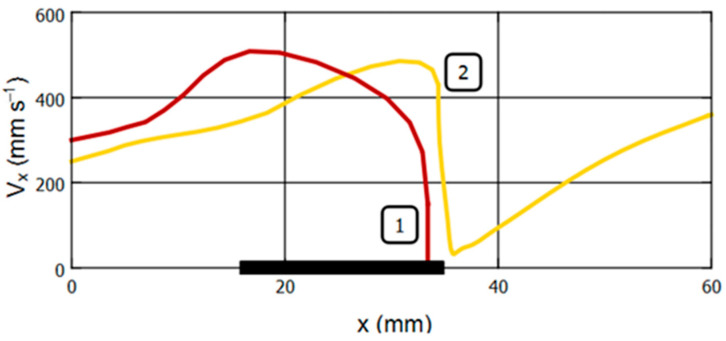
Computer simulation of the linear velocity of the ferrogel bead in the tube at fluid flow using the model (the model description is presented in Section 4.5; see also Supplementary Videos S3 and S4). Tracks 1 and 2 correspond to the magnetic field strengths at the surface of the EM core of 40 and 32 kA/m, respectively. The origin of the coordinates corresponds to the beginning of the observation. The thick horizontal line at the X-axis represents the position of the electromagnet core.

Figure 12 and Figure 13 show the simulation results of ferrogel bead mechanical behavior at experimental conditions that correspond to the data in Videos S1 and S2, Figure 9 and Figure 10. Videos S3 and S4 in the Supplementary Materials contain visual demonstrations of the simulations obtained.

Comparison of the curves obtained by modeling with the experimental ones shows good qualitative agreement for both the trajectories and the velocity projection. As the key results, we can consider the reproduction of the capture of the ferrogel bead by a magnet in case “1” and the unstable capture with subsequent detachment of the ferrogel bead in case “2”. Such detachment is accompanied by oscillations of the gel sample due to pulsations of the hydrodynamic flow near it and, as a consequence, oscillations of the hydrodynamic force. This effect was also observed in the experimental studies.

During simulations, the ferrogel bead, entering the zone of the magnetic field, began to be attracted to the center of the EM core, as a result of which it accelerated mainly along the tube. As it moves, the horizontal component *Vx* of the magnetic force decreases while the vertical component *Vy* increases, and the bead rapidly moves toward EM until it touches the tube wall. In the experimental study, this effect was also observed, but its severity depends significantly on the initial state of the system.

Quantitative differences between the experimental results and computer simulations can be explained by the use of a simple simulation model that does not take into account the rotation of the gel around its axis, the inhomogeneity and asymmetry of the magnetic field, as well as natural velocity fluctuations in the fluid flow. It is possible to take these factors into account, but it will require a much more detailed study of the mechanical properties of a spherical ferrogel bead sample and the design and fabrication of a more precise source of the magnetic field. In addition, to accurately determine the boundary conditions, it is necessary to control the hydrodynamic characteristics of the liquid in the measurement area with high accuracy and resolution. All this requires complex tools and further development of experimental and simulation methods.

## 3. Conclusions

In this work, the possibility of positioning a spherical magnetic alginate ferrogel bead using a magnetic field was analyzed both experimentally and using computer simulations. Spherical beads of composite ferrogels based on a physical network of calcium alginate filled with a 12.6% weight fraction of magnetite particles sized 200–300 nanometers in diameter. The experimentally studied physical properties of the ferrogel determine its significant magnetic response. Thus, even when the sphere is in a tube with an intense fluid flow, the magnetic force of attraction is sufficient to capture and hold the sample. This effect has been demonstrated experimentally. However, it was shown that the trajectory, the speed of the ferrogel bead, and, consequently, the nature of the interaction of the sample with the magnet are significantly affected by the hydrodynamic force. This force arises as a result of the complex combination of fluid flow in the tube and the simultaneous movement of the liquid around the bead. As a result, the hydrodynamic force tends not only to move the ferrogel bead along the tube but also to keep it at an equal distance from its walls. Together with the contribution of the magnetic force, this leads to an uneven movement of the spherical sample along a complex trajectory, in which it becomes difficult to predict the capture of the sample by the magnet.

The developed computer model takes into account both the hydrodynamic and magnetic components of the forces acting on the sphere in the fluid flow in the tube. The carried-out simulations qualitatively reproduce the experimental results obtained. At the same time, when comparing the data from computer simulations and experimental studies, one should note quantitative discrepancies. The key reason for these discrepancies seems to be the inhomogeneities of both the magnetic field and the parameters of the medium. Obviously, these inhomogeneities will be decisive if precise positioning of the ferrogel is required.

Finally, the results presented in this study on the remote positioning of a spherical biocompatible platform can be useful in designing delivery systems for medicinal substances. In fact, the paper mainly discusses the principles of sample capture and attachment to the delivery site in fluid media. It turned out that the estimation of the attractive force by Equation (6) for ferrogel beads and cylinders [33] does not critically depend on the shape and size of the samples. The universality of the proposed approach implies the possibility of scaling the effects of FG attraction to a magnet based on knowledge of the magnetic susceptibility of the material and the features of the spatial distribution of the magnetic field. On the other hand, the contribution of the hydrodynamic force to the positioning of the delivery system depends significantly on the shape and size of the sample, its velocity in the flow, and the properties of the medium. Within the framework of a simple hydrodynamic model, this contribution can only be predicted qualitatively. At the same time, the simulation results show how mathematical model development can increase the accuracy of predictions. Further research in this direction is needed.

## 4. Materials and Methods

### 4.1. Synthesis of Ferrogel Beads

In the synthesis of FGs [18], first a stock 5% water solution of sodium alginate, purity ≥99.5% (NaAlg) (Sigma-Aldrich, St. Louis, MO, USA), was prepared under permanent stirring. The molecular mass of NaAlg obtained by viscometry in 0.1 M NaCl was 190.5 kDa (with Mark–Houwink constants *K* = 0.023, *a* = 0.984 [41,42,43]. Then, 10% (wt.) of magnetite particles were added to the stock solution and vigorously stirred. Particles did not sediment from the suspension due to its viscosity.

This suspension was manually added dropwise using a 5G syringe with a 0.5 mm needle into an aqueous solution of 0.5 M calcium chloride, purity >99% (CaCl_2_·2H_2_O) (Himreact, Klin, Moscow region, Russia). Due to the Na^+^/Ca^2+^ ionic exchange, the droplets immediately precipitated as elastic ferrogel beads, as the CaAlg polymer is insoluble in water. The FG-beads were kept in saline solution for 2 days for the completion of ion exchange. To achieve equilibrium swelling, FG-beads were washed in distilled water for 7 days with daily water renewal. As a reference, the blank CaAlg gels were prepared under the same conditions but without the addition of magnetite to the stock solution of NaAlg.

The average diameter of gel beads was determined with the use of a digital micrometer thickness gauge (Weihai Minghui Measuring Tool Co., Ltd., Weihai, China) and also estimated using optical microscopy.

### 4.2. Methods for Magnetic Particles and Ferrogels Characterization

Transmission electron microscopy (TEM) studies of used magnetic particles were done using the JEOL JEM2100 instrument (JEOL Corporation, Tokyo, Japan) for the samples spread onto conventional carbon-conductive substrates. Optical microscopy observations of the surface of a single FG spherical bead were made using an optical microscope Nicon L-UEPI (Boston Industries Inc., Boston, MA, USA). The geometry of the experiment is described in Figure 1c. A single FG bead is placed on a glass surface with a small drop (of the order of 5 mg) of water in order to keep the size of the bead constant during observations. The X-ray diffraction (XRD) studies were performed with the use of the Discover diffractometer D8 Discover (Bruker Corporation, Billerica, MA, USA), operating at 40 kV and 40 mA at Cu-K*α* radiation (*λ* = 1.5418 Å), with a graphite monochromator and a scintillation detector. The corresponding intensities were measured in the standard Bragg–Brentano geometry for 2θ° angles from 15 to 90° with a step of 0.02°. Bruker software TOPAS-3 with Rietveld full-profile refinement was employed for the quantitative analysis of the diffractograms.

Saturation magnetization of magnetic particles (*Ms*) was determined by vibration sample magnetometry (VSM) (Cryogenics, Ltd. VSM, London, UK). The content of magnetite in FG-beads was determined by thermal analysis: thermogravimetry/differential scanning calorimetry/mass spectrometry. Thermal decomposition of previously dried FG samples was done by their heating from 40 to 1000 °C at a rate of 10 K/min in the air using a NETSCH STA403 thermal analyzer with a built-in quadrupole mass spectrometer (NETZSCH Geratebau Gmbh, Selb, Germany).

The equilibrium swelling ratio, or, in other words, the maximal water uptake of CaAlg/Fe_3_O_4_ ferrogel (FG) and blank CaAlg hydrogel (HG), was determined gravimetrically. About 50 ferrogel or hydrogel beads were weighed and then dried in an oven at 70 °C to a constant weight. The dry residue was weighed again, and the swelling ratio was calculated according to Equation (1).

Magnetic measurements were made using a vibrating sample magnetometer at room temperature. The magnetic hysteresis loops M(H) were measured by VSM. The primary magnetization curve was also obtained for both particles and the FG-bead.

### 4.3. Experimental Setup for the Measurement of Attractive Force of Ferrogel Beads to a Magnet

**Figure 14:**
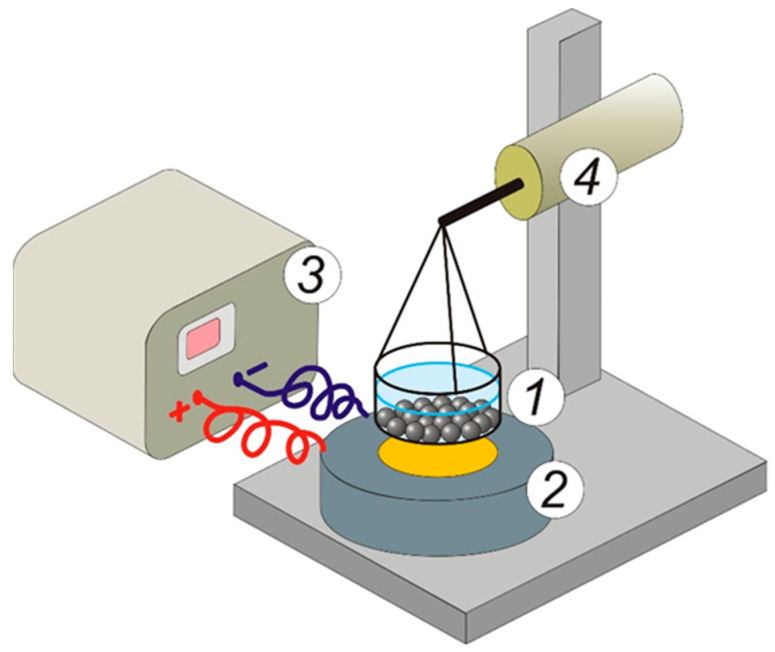
Schematic sketch of the experimental setup for the measurement of the attractive force of ferrogel beads on a magnet. 1—cuvette filled with water and beads; 2—EM; 3—DC power supply; 4—FT. See explanation in the text.

The experimental setup (Figure 14) for measuring the mechanical force acting on a ferrogel in a nonuniform magnetic field was described in detail earlier [33]. Briefly, a force transducer (FT) was fixed on a massive tripod. A thin-walled polystyrene cuvette with a diameter of 18.6 mm was mechanically connected to the FT using rigid copper braces. The FT accuracy (output RMS value of the noise) was 0.03 mN. The cuvette was filled with distilled water and contained one layer of ferrogel beads (36 samples). A commercial electromagnet (EM) CL–34/18 (Cinlin, Guangzhou, China) was positioned under the cuvette coaxially to the sensitive lever of FT at a distance of 4 mm from the surface of the EM core (diameter 18 mm).

The magnetic field strength of EM was provided by a stabilized power supply in the range from 0 to 15 V, which corresponded to 0–43 kA/m at the surface of the EM core. The dependence of the magnetic field strength (*H*) on the distance from the EM surface (*z*, in mm) was determined as follows: *H* = *H*_0_ (1 − 0.073z), where *H*_0_ is the field strength at the surface.

The control experiments with blank CaAlg gels without MPs were done to check the effect of EM on the FT. The test was successfully passed at any strength of the magnetic field.

### 4.4. Experimental Setup for the Modeling of Ferrogel Bead Motion in a Fluid Flow in the Presence of a Magnetic Field

**Figure 15:**
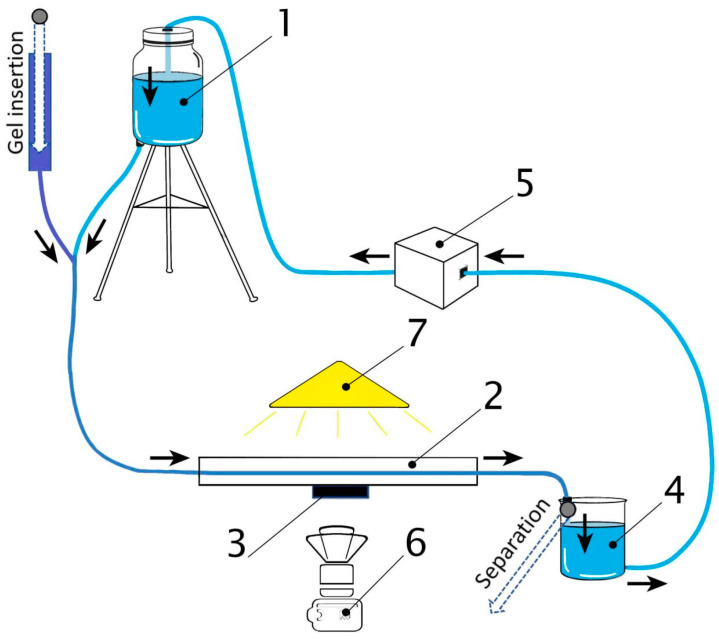
Schematic of experimental hydrodynamic setup for positioning ferrogel beads in a fluid flow. 1—main reservoir mounted on a tripod with adjustable height, 2—straight section of the tube fixed on a horizontal platform, 3—electromagnet connected to a power supply (not present), 4—buffer reservoir, 5—peristaltic pump, 6—video camera, 7—light source. See explanation in the text.

Figure 15 schematically shows the main elements of an experimental hydrodynamic setup for positioning ferrogel beads in a fluid flow. The system contains the main glass reservoir for liquid (distilled water), mounted on a tripod with adjustable height. The reservoir outlet was connected to a silicone tube with an inner diameter of 8.0 mm and a wall thickness of 1.5 mm. A straight section of the tube, 1 m long, was fixed on a horizontal wooden platform (40 mm thick) using flexible clamps. The electromagnet was placed inside the platform body so that its core surface was in direct contact with the outer wall of the tube (see Figure 7). An electromagnet CL–34/18 was used, i.e., the same as in experiments on the measurements of beads’ attractive force.

The water from the tube flowed into the buffer reservoir, from which it was returned back to the main reservoir by means of a BT100FJ precision peristaltic pump with adjustable output (LeadFluid, Guangzhou, China). In the area of the magnet, the lens of a high-speed video camera (Sony ZV-1, Beijing, China) was mounted on the side perpendicular to the long axis of the tube. A light source with an adjustable wavelength was installed above the magnet area to obtain the best video image contrast. The frame rate for video recording was 1000 fps. Video recordings of the motion of a gel sample in a magnetic field are presented in the Supplementary Files with a 40 times slowdown during playback.

The ferrogel bead was immersed in the main reservoir, and then it was moved by the water flow through the tube (see Videos S1 and S2). A sieve was installed in the buffer reservoir to prevent the circulation of beads through the pump. The fluid flow rate was set by the height of the water column in the main reservoir. The required value of the height of the water column was maintained constant by adjusting the speed of the pump rollers. Thus, the volumetric flow rate of water in the hydrodynamic system was equal to the pump capacity.

### 4.5. Mathematical Model of Ferrogel Bead Motion in a Flow at the Presence of a Magnetic Field

A model of a gel sample in the magnetic field is implemented in the FlowVision (Tesis Ltd., Moscow, Russia) software package with the use of the moving body algorithm [44].

The fluid flow is represented by a continuous laminar medium, the dynamics of which could be expressed by equations:

$$\begin{array}{ll} & \nabla \cdot V=0,\\ & \rho \frac{\partial V}{\partial t}+\rho \left(V\cdot \nabla \right)V=-\nabla P+\nabla \cdot \tau_{e}+F_{grav},\\ & \tau_{e}=\mu \left(2S-\frac{2}{3}(\nabla \cdot V)I\right),\;F_{grav}=-mg,\\ & \;S_{ij}=\frac{1}{2}\left(\frac{\partial V_{i}}{\partial x_{j}}+\frac{\partial V_{j}}{\partial x_{i}}\right). \end{array}$$

Here, V is the velocity, t is a time, ρ is the constant density of the liquid, P is the pressure, $\tau_{e}$ is the tensor of viscous stresses, m is a mass, g—is gravitational acceleration, μ is the coefficient of dynamic viscosity, and I represents the unit tensor.

**Figure 16:**
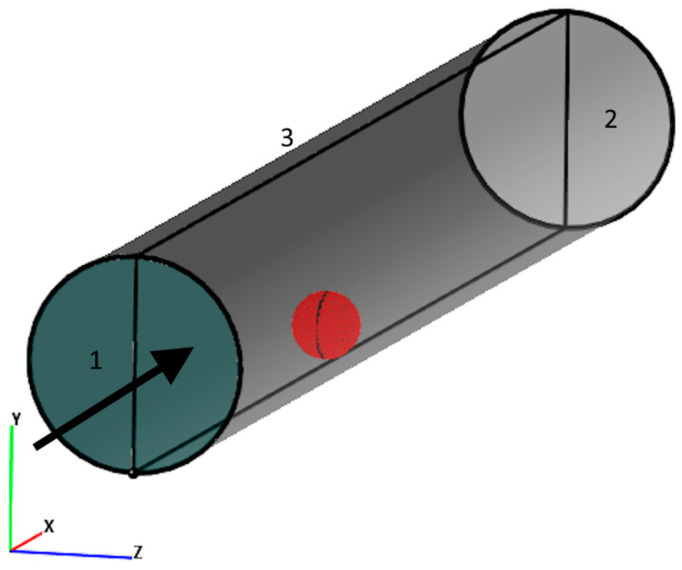
Scheme of the computational domain and the used boundary conditions. Inflow boundary “1” defines a fluid flow with a fixed velocity, flowing in a cylindrical domain with rigid walls “3”. The flow interacts with the ball and exits through outflow boundary “2” defined by zero pressure gradient.

Computer simulations of the gel sample motion in the cylindrical domain (Figure 16) were performed considering the following boundary conditions: at the inflow boundary “1”—constant flow velocity along the normal to the boundary was 0.32 m/s, and at the outflow boundary “2”—zero-gradient condition for pressure was set. Boundary “3” was defined as an incompressible wall where the non-slip condition for the velocity was satisfied. The problem was solved in a symmetric formulation with respect to the XY-plane. A sample in the domain is highlighted in red, which is a surface with a similar boundary 3 wall condition. At the initial moment, the sample is located near boundary 1 at a height of 0.003 mm. Hereinafter, the coordinates of the bead are the coordinates of its center.

**Figure 17:**
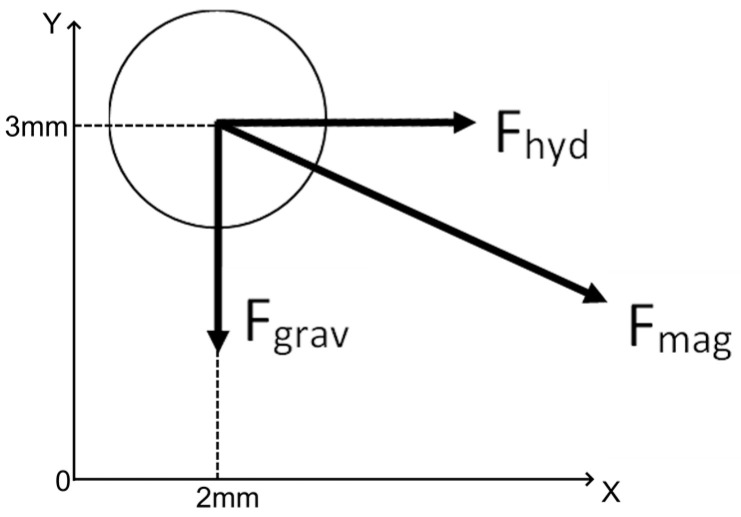
Scheme of forces acting on the gel sample and its initial position in the computational domain.

Three forces are constantly acting on the ball: the gravity force $F_{\mathrm{grav}}$, the force of interaction with the fluid flow–hydrodynamic force $F_{\mathrm{hyd}}$ and the force $F_{\mathrm{mag}}$ of magnetic attraction to the magnet (Figure 17).

At the initial moment of time, the velocity of the sample along the *X*-axis $V_{0}\;$= 0.3 m/s for the case of holding the sample at 40 kA/m at the core of EM and $V_{0}\;$= 0.25 m/s for the case of 32 kA/m at the core of EM.

The hydrodynamic force is described by the equation:

$$F_{\mathrm{hyd}}={∯}_{Sb}PndSb-{∯}_{Sb}\;\mu \;\frac{\partial V_{\tau }}{\partial n}dSb,$$

where Sb—surface of the ball, *n*—normal to the surface, $V_{\tau }$—tangential component of velocity.

The magnetic force $F_{\mathrm{mag}}$ is determined using Equation (6). The parameters used for the simulations are presented in Table 2.

## Figures and Tables

**Table 1:** Selected characteristics of CaAlg ferrogel beads.

Sample	Integral Swelling Ratio, *a*	Weight Loss in TG (%)	Weight Fraction of Magnetite in Dry Gel, *γ*	Swelling Ratio of Polymer Matrix, *a*’	Weight Fraction of Magnetite in FG Bead, w (%)	Magnetic Susceptibility of FG
HG	14.4	86.32	-	14.4	-	
FG	4.5	26.07	0.698	15.0	12.6	0.13

**Table 2:** Simulation parameters.

Parameter	Description	Value
μ, Pa s	Coefficient of dynamic viscosity	0.00102
g, m s^−2^	Gravitational acceleration	9.8
d, m	Gel sample diameter	0.00238
ρ, kg/m^−3^	Fluid density	1000
$\rho_{gel}$, kg/m^−3^	Gel sample density	1110

## Data Availability

Not applicable.

## Supplementary Materials

The following supporting information can be downloaded at: https://www.mdpi.com/article/10.3390/gels9090711/s1, Video S1: Bead motion in a flow at 32 kA/m (experiment); Video S2: Bead motion in a flow at 40 kA/m (experiment); Video S3: Bead motion in a flow at 32 kA/m (simulation); Video S4: Bead motion in a flow at 40 kA/m (simulation).
